# SLEEP QUALITY, DURATION, AND EFFICIENCY IN LOW-INCOME OLDER ADULTS WITH AND WITHOUT DISABILITIES

**DOI:** 10.1093/geroni/igae098.1180

**Published:** 2024-12-31

**Authors:** James Brightman, Kworweinski Lafontant, Jethro Raphael Suarez, Jennifer Crook, Ladda Thiamwong

**Affiliations:** University of Central Florida, Orlando, Florida, United States; University of Central Florida, Orlando, Florida, United States; University of Central Florida; University of Central Florida, Orlando, Florida, United States; University of Central Florida, Orlando, Florida, United States

## Abstract

Seventy million Americans experience chronic sleep disorders and older adults are more likely than younger ones to have problems sleeping. Sleep is a critical part to the circadian rhythm, and getting adequate sleep can help prevent falls and increase physical functioning. Even though sleep quality declines as a function of normal aging, the presence of a physical disability may exacerbate that natural decline. Income status can also impact sleep quality and the prevalence of dysregulated sleep may lead to an increased use of medicinal sleep aids to alleviate the issue. The chronic use of sleep aids, however, may not be beneficial for alleviating sleep complaints. In this study, we assessed the sleep quality and sleep aid use of 87 low-income community-dwelling older adults with (n = 24) and without (n = 63) a physical disability. Sleep quality, duration, and efficiency were assessed subjectively with the Pittsburgh Sleep Quality Index. Sleep duration and efficiency were objectively measured with actigraphy. Participants self-reported medicinal sleep aid usage. Significant group differences were observed in sleep duration measured objectively (p = 0.02). No group differences were identified in subjective sleep quality factors (p = 0.21) or medicinal sleep aid use (p = 0.41). Our findings show physical disability may be a factor in sleep duration, though neither associated with worsened sleep perception nor greater reliance on medicinal sleep aids. Implications of our research highlight the importance of using subjective and objective measures when assessing sleep quality and carefully considering the use of medicinal sleep aids.

